# Polymorphisms of shadow of prion protein gene (*SPRN*) in Korean native cattle (Hanwoo) and Holstein cattle

**DOI:** 10.1038/s41598-020-72225-x

**Published:** 2020-09-17

**Authors:** Yong-Chan Kim, Seon-Kwan Kim, Sae-Young Won, Byung-Hoon Jeong

**Affiliations:** 1 Korea Zoonosis Research Institute, Jeonbuk National University, 820-120, Hana-ro, Iksan, Jeonbuk 570-390 Republic of Korea; 2 Department of Bioactive Material Sciences and Institute for Molecular Biology and Genetics, Jeonbuk National University, Jeonju, 561-756 Republic of Korea

**Keywords:** Genetics, Computational biology and bioinformatics, Genotype, Haplotypes

## Abstract

Bovine spongiform encephalopathy (BSE) is a fatal infectious neurodegenerative disease caused by the accumulation of pathogenic prion protein (PrP^Sc^) in the central nervous system (CNS), particularly in the brain. In a recent study, the shadow of prion protein (Sho), encoded by the shadow of prion protein (*SPRN*) gene, accelerates the progression of prion diseases, and a 12-bp insertion/deletion polymorphism in the coding region of the *SPRN* gene is associated with susceptibility to atypical BSE-affected Polish cattle. To date, the genetic study of the *SPRN* gene in Korean cattle has not been performed. In this study, we investigated the genotype and allele frequencies of *SPRN* polymorphisms in 235 Hanwoo and 212 Holstein cattle and analyzed the linkage disequilibrium (LD) and haplotypes of *SPRN* polymorphisms. In addition, we compared the distribution of the 12-bp insertion/deletion polymorphism between atypical BSE-diagnosed Polish cattle and Korean cattle to evaluate the susceptibility of atypical BSE. Furthermore, we estimated a deleterious effect of polymorphisms on the Sho protein using PROVEAN. We found a total of seven polymorphisms, including one novel single nucleotide polymorphism (SNP), c.231G>A. We also found significantly different distributions of genotype, allele and haplotype frequencies of seven polymorphisms between Hanwoo and Korean Holstein cattle. In addition, all polymorphisms showed strong LDs among the seven polymorphisms. Interestingly, Hanwoo cattle showed more potential susceptible distribution in the genotype and allele frequencies of the 12-bp insertion/deletion polymorphisms of the *SPRN* gene than Holstein cattle. Finally, using PROVEAN, we found one novel deleterious nonsynonymous SNP to Sho protein, c.110G>C (G37A). To the best of our knowledge, this is the first study of the *SPRN* gene in Korean cattle.

## Introduction

Transmissible spongiform encephalopathies (TSEs) are infectious neurodegenerative diseases in humans and animals that are associated with the accumulation of abnormal prion protein (PrP^Sc^) originating from the structural conversion of normal prion proteins (PrP^C^) in brain tissue^1,2^. Bovine spongiform encephalopathy (BSE) belongs to TSEs, which also include scrapie in sheep and goats, chronic wasting disease (CWD) in elk and deer, transmissible mink encephalopathy (TME) in mink, feline spongiform encephalopathy (FSE) in cats, cheetah and pumas, and Creutzfeldt–Jakob disease (CJD), fatal familial insomnia (FFI) and Gerstmann–Sträussler–Scheinker syndrome (GSS) in humans. The origin of BSE is still unclear; however, it has been postulated that BSE-infected cattle are sporadic or infected through the meat and bone meal from scrapie-affected sheep^3–16^. BSE was first discovered in the UK in 1985, and consumption of BSE-infected meat is a major cause of variant CJD in humans^17–19^ In cattle, although several single nucleotide polymorphisms (SNPs) have been reported in the open reading frame (ORF) of the prion protein gene (*PRNP*), these SNPs showed no significant correlations with BSE susceptibility. However, only a 23-bp insertion/deletion polymorphism in the promoter region and a 12-bp insertion/deletion polymorphism in intron 1 of the *PRNP* gene are associated with the susceptibility of BSE^20,21^. However, although gene-regulated polymorphisms play pivotal role in several diseases, the insertion/deletion polymorphisms of the *PRNP* gene only modulate the expression level of the *PRNP* gene by mediating the binding ability of transcription factors, including RP58 and SP1, and they do not induce misfolding of the prion protein^22^. Thus, several studies have tried to find novel candidate transcription factors using association analyses and quantitative trait loci (QTL) mapping, and it has been suggested that genetic resistance or susceptibility to BSE is able to be controlled by genomic regions other than the *PRNP* locus^23,24^. Previous studies have reported that polymorphisms of the shadow of prion protein gene (*SPRN*) were associated with susceptibility to CJD, scrapie and BSE. In humans, the null allele of the *SPRN* gene showed association with variant CJD^25^. In sheep, a polymorphism causing a deletion of two alanines was related to the susceptibility for scrapie^26^. In goats, an insertion/deletion polymorphism located on the 3′ UTR was associated with the susceptibility for scrapie^27^. In cattle, a 12-bp insertion/deletion polymorphism causing a deletion of 4 amino acids (67_70delAAAG) in the repetitive alanine-rich sequence of the *SPRN* gene was found in only one case of L-type atypical BSE in Polish cattle^28^. Although the association of prion diseases with polymorphisms in the *SPRN* gene has apparently existed, genetic studies of the *SPRN* gene in Korean cattle, including Hanwoo and Holstein cattle, have not been performed thus far. In Korea, Hanwoo is representative native breed for beef production and Holstein is major commercial breed for milk production (https://kosis.kr).

In the present study, we investigated the genotype and allele frequencies of *SPRN* polymorphisms in Hanwoo and Korean Holstein cattle. In addition, we analyzed the linkage disequilibrium (LD) and haplotypes of *SPRN* polymorphisms. Furthermore, we compared the distributions of the 12-bp insertion/deletion polymorphism between atypical BSE-diagnosed Polish cattle and Korean cattle. Finally, we evaluated a deleterious effect of polymorphisms causing protein sequence changes in the shadow of prion protein (Sho) using PROVEAN (https://provean.jcvi.org/seq_submit.php)^29,30^.

## Results

### Investigation of polymorphisms of the *SPRN* gene in Korean cattle

**Figure 1:**
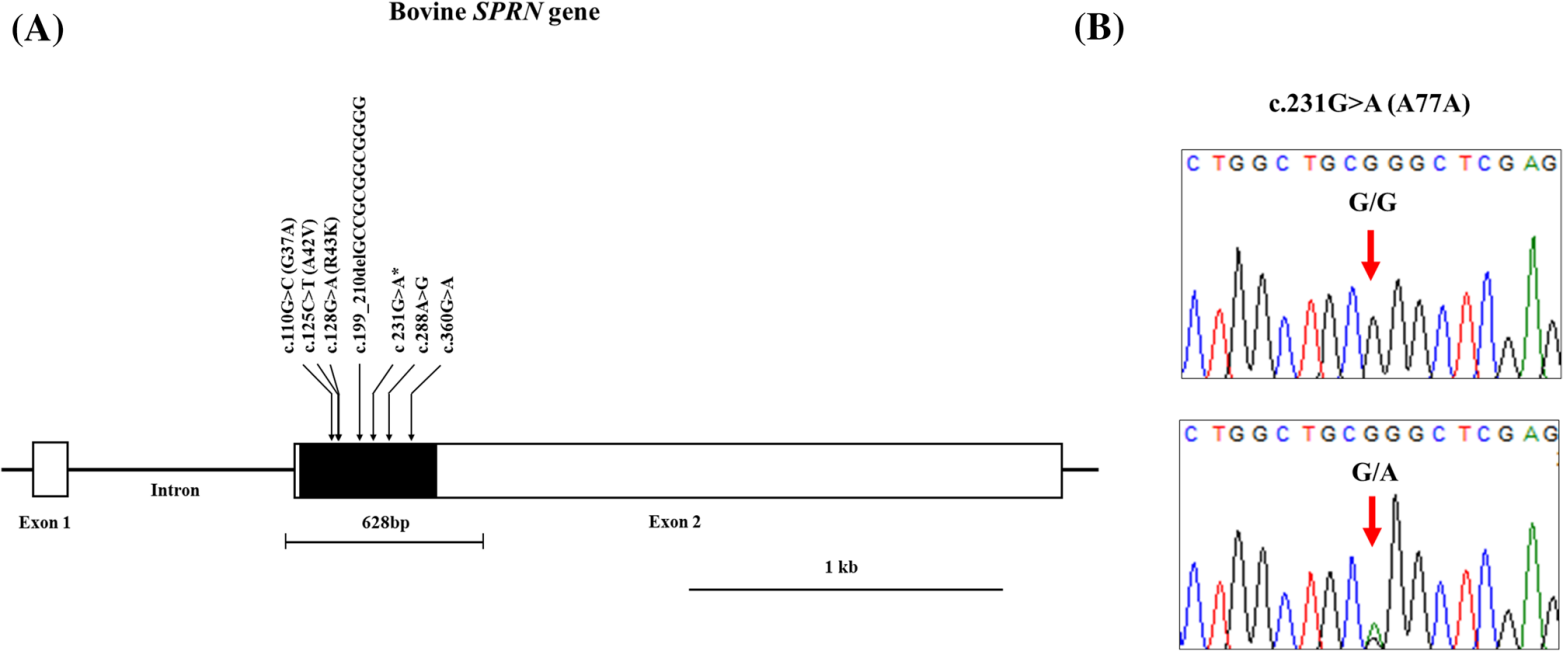
(**A**) Gene map and polymorphisms identified in the shadow of prion protein gene (*SPRN*) on chromosome 26 in Korean cattle. The open reading frame (ORF) within exon 2 is marked by a shaded block, and the 5′ and 3′ untranslated regions (UTRs) are indicated by a white block. The arrows indicate the seven polymorphisms found in this study. The edged horizontal bar indicates the region sequenced. Asterisks denote novel single nucleotide polymorphisms (SNPs). (**B**) Electropherogram of novel SNPs in the *SPRN* gene. colors indicate individual bases of the DNA sequence using an ABI 3730 automatic sequencer (blue: cytosine; red: thymine; black: guanine; and green: adenine).

To investigate the genotype and allele frequencies of *SPRN* gene polymorphisms in Hanwoo and Holstein cattle, we performed direct sequencing of the *SPRN* gene in 235 Hanwoo and 212 Holstein cattle. The amplicon composed of 628 bp including ORF (432 bp) was confirmed by agarose gel electrophoresis (Supplementary Figs. 1, 2) and was homologous with the *SPRN* gene of *Bos taurus* that is registered in GenBank (Gene ID: 616266). We performed the quality check on sequencing results using phred quality score by CodonCode Aligner 9.0.1 (CodonCode Corporation, United Kingdom) and genotyping of each nucleotide with Q > 40 (Supplementary Figs. 3, 4). We found a total of seven polymorphisms, including 3 nonsynonymous SNPs [c.110G>C (G37A), c.125C>T (A42V) and c.128G>A (R43K)], three synonymous SNPs [c.231G>A (A77A), c.288A>G (E96E) and c.360G>A (G120G)] and 1 insertion/deletion polymorphism [c.199_210delGCCGCGGCGGGG (67_70delAAAG)] in the coding region (Fig. 1A). Among the seven polymorphisms, c.231G>A (A77A) is a novel SNP found in this study (Fig. 1B). Detailed information on the genotype and allele distributions of the seven polymorphisms is described in Table 1. In brief, except for c.360G>A (G120G) in Holstein cattle, all other polymorphisms were in HWE. One SNP (c.110G>C) and one insertion/deletion polymorphism (c.199_210delGCCGCGGCGGGG) were found in only Hanwoo cattle, and two SNPs (c.125C>T and c.128G>A) were found in only Holstein cattle. Notably, the genotype and allele distributions of three polymorphisms (c.288A>G, c.360G>A and c.199_210delGCCGCGGCGGGG) were significantly different between Hanwoo and Holstein cattle (Table 1).

**Table 1:** Genotype and allele frequencies of *SPRN* polymorphisms in Korean cattle.

Polymorphisms	Breeds	Total, n	Genotype frequency, n (%)			*P* value	Allele frequency, n (%)		*P* value	HWE
**c.110G>C (G37A)**			G/G	G/C	C/C		G	C		
	Hanwoo	235	234 (99.5)	1 (0.5)	0 (0)	1.0	469 (99.8)	1 (0.2)	1.0	0.973
	Holstein	212	212 (100)	0 (0)	0 (0)		424 (100)	0 (0)		–
**c.125C>T (A42V)**			C/C	C/T	T/T		C	T		
	Hanwoo	235	235 (100)	0 (0)	0 (0)	0.1059	470 (100)	0 (0)	0.1063	–
	Holstein	212	209 (98.6)	3 (1.4)	0 (0)		421 (99.3)	3 (0.7)		0.917
**c.128G>A (R43K)**			G/G	G/A	A/A		G	A		
	Hanwoo	235	235 (100)	0 (0)	0 (0)	0.1059	470 (100)	0 (0)	0.1063	–
	Holstein	212	209 (98.6)	3 (1.4)	0 (0)		421 (99.3)	3 (0.7)		0.917
**c.199_210delGCCGCGGCGGGG (67_70delAAAG)**			WT/WT	WT/Del	Del/Del		WT	Del		
	Hanwoo	235	214 (91)	21 (9)	0 (0)	**< 0.0001**	449 (95.5)	21 (4.5)	**< 0.0001**	0.473
	Holstein	212	212 (100)	0 (0)	0 (0)		424 (100)	0 (0)		–
**c.231G>A (A77A)**			G/G	G/A	A/A		G	A		
	Hanwoo	235	231 (98.3)	4 (1.7)	0 (0)	0.3855	466 (99.1)	4 (0.9)	0.3769	0.895
	Holstein	212	211 (99.5)	1 (0.5)	0 (0)		423 (99.8)	1 (0.2)		0.972
**c.288A>G (E96E)**			A/A	A/G	G/G		A	G		
	Hanwoo	235	62 (26.4)	123 (52.3)	50 (21.3)	**0.0007**	247 (52.5)	223 (47.5)	**0.0004**	0.447
	Holstein	212	92 (43.4)	88 (41.5)	32 (15.1)		272 (64.2)	152 (35.8)		0.155
**c.360G>A (G120G)**			G/G	G/A	A/A		G	A		
	Hanwoo	235	213 (90.6)	22 (9.4)	0 (0)	**0.0001**	448 (95.3)	22 (4.7)	**< 0.0001**	0.451
	Holstein	212	169 (79.7)	33 (15.6)	10 (4.7)		371 (87.5)	53 (12.5)		**< 0.001**

Bold texts indicate P < 0.05.

### LD analysis of polymorphisms of the bovine *SPRN* gene

We examined whether there was a strong linkage disequilibrium (LD) among the seven polymorphisms. Except for the LD between the c.360G>A SNP and the c.288A>G (D′ = 0.881), all other polymorphisms showed strong LDs with D′ values > 0.9 (Table 2).

**Table 2:** Linkage disequilibrium (LD) analysis of *SPRN* polymorphisms in Korean cattle.

	c.110G>C	c.125C>T	c.128G>A	c.199_210del	c.231G>A	c.288A>G	c.360G>A
c.110G>C	–	1.0	1.0	1.0	1.0	1.0	1.0
c.125C>T	–	–	1.0	1.0	1.0	1.0	1.0
c.128G>A	–	–	–	1.0	1.0	1.0	1.0
c.199_210del				–	1.0	1.0	0.982
c.231G>A	–	–	–		–	1.0	1.0
c.288A>G	–	–	–		–	–	0.881
c.360G>A	–	–	–		–	–	–

### Haplotype analysis of polymorphisms in the bovine *SPRN* gene

We investigated the haplotype distributions of seven polymorphisms in the *SPRN* gene. Five major haplotypes of *SPRN* gene polymorphisms were found in Korean cattle, and the GCGWtGAG haplotype was most frequently observed in Hanwoo (53.0%) and Holstein cattle (63.2%). Detailed information on haplotype frequencies in Korean cattle is described in Table 3. Notably, the distributions of all haplotypes were significantly different between Hanwoo and Holstein cattle (Table 3).

**Table 3:** Haplotype analysis of *SPRN* polymorphisms in Korean cattle.

Haplotypes	Frequency		P value
	Hanwoo (n = 470)	Holstein (n = 424)	
GCGWtGAG	249 (0.530)	268 (0.632)	0.002
GCGWtGGG	173 (0.368)	100 (0.236)	< 0.0001
GCGWtGGA	22 (0.047)	48 (0.113)	0.0002
GCGDelGGG	21 (0.045)	0 (0.000)	< 0.0001
GCGWtGAA	0 (0.000)	5 (0.012)	0.0237
Others^a^	5 (0.010)	5 (0.012)	–

^a^Contain rare haplotypes with frequency < 0.01.

### Comparison of distribution of indel polymorphism in Korean cattle

**Figure 2:**
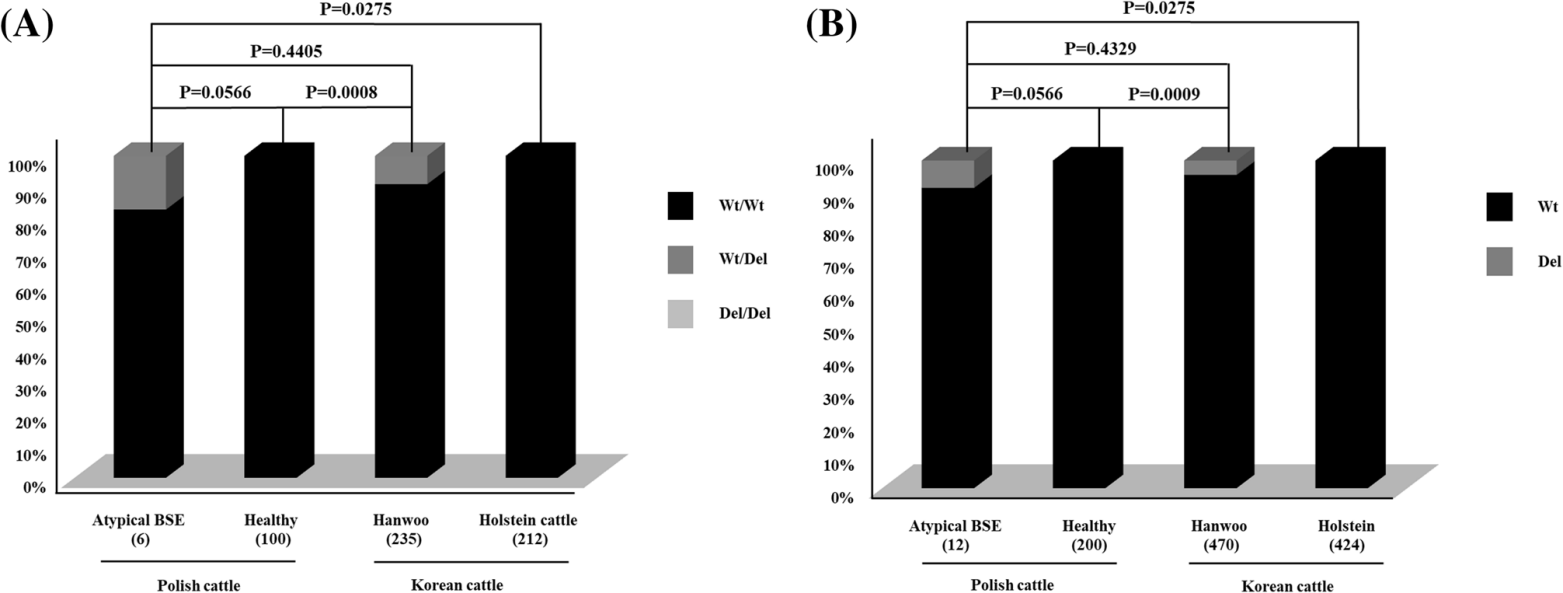
(**A**) Comparison of genotype frequencies on the c.199_210delGCCGCGGCGGGG (67_70AAAG) insertion/deletion polymorphism of the shadow of prion protein (*SPRN*) gene in atypical BSE-affected Polish cattle and Korean cattle. (**B**) The comparison of allele frequencies on the c.199_210delGCCGCGGCGGGG (67_70AAAG) insertion/deletion polymorphism of the shadow of prion protein (*SPRN)* gene in atypical BSE-affected Polish cattle and Korean cattle. Parentheses indicate the number of cattle.

A previous study reported that the c.199_210delGCCGCGGCGGGG polymorphism showed a significantly different distribution between healthy and atypical BSE-affected Polish cattle. To compare the distribution of indel polymorphism in Korean cattle, we compared the genotype and allele distribution of the c.199_210delGCCGCGGCGGGG polymorphism between atypical BSE-affected and Korean cattle (Fig. 2). Interestingly, the genotype distribution of the c.199_210delGCCGCGGCGGGG polymorphism was significantly different between BSE-affected Polish cattle and Holstein cattle (P = 0.0275). However, the genotype distribution of the c.199_210delGCCGCGGCGGGG polymorphism was not significantly different between BSE-affected Polish cattle and Hanwoo cattle (P = 0.4405). The allele distribution of the c.199_210delGCCGCGGCGGGG polymorphism was significantly different between BSE-affected Polish cattle and Holstein cattle (P = 0.0275). However, the allele distribution of the c.199_210delGCCGCGGCGGGG polymorphism was not significantly different between BSE-affected Polish and Hanwoo cattle (P = 0.4329).

### Evaluation of the deleterious effect of polymorphisms on the Sho protein

To estimate the impact of polymorphisms causing protein sequence changes in the *SPRN* gene, we utilized the PROVEAN program. c.110G>C (G37A) and c.199_210delGCCGCGGCGGGG (67_70delAAAG) polymorphisms were predicted as being “deleterious” with scores of − 4.635 and − 11.067, respectively. However, c.125C>T (A42V) and c.128G>A (R43K) SNPs were predicted as being “neutral” with scores of − 1.890 and − 2.377, respectively (Table 4).

**Table 4:** In silico evaluation of the impact of *SPRN* polymorphisms on the Sho protein.

Variants	Score	Prediction (cutoff = − 2.5)
**c.110G> C**		
G37A	− 4.635	Deleterious
**c.125C> T**		
A42V	− 1.890	Neutral
**c.128G> A**		
R43K	− 2.377	Neutral
**c.199_210delGCCGCGGCGGGG**		
67_70delAAAG	− 11.067	Deleterious

## Discussion

BSE is classified into two types: classical and atypical BSE. Atypical BSE is also further classified into two types according to the molecular pattern of PrPres with a protease-resistant core of lower (L-type) or higher (H-type) molecular mass^31^. In Korea, none of the types of BSE have been reported thus far; however, BSE-related genetic factors, including the SNPs 4138 and 13861, a 12-bp insertion/deletion polymorphism in the Intron 1, a 23-bp insertion/deletion polymorphism in the promoter region and the allele E211K germline mutation in the *PRNP* gene, have been investigated and their susceptibilities have been evaluated by comparing the genetic distribution of BSE-related polymorphisms between BSE-affected animals and Korean cattle^11,20,32^. However, the insertion/deletion polymorphisms of the *SPRN* gene, previously reported as the atypical L-type BSE-related genetic factor, have not been investigated in Korean cattle thus far^28^. In the present study, for the first time, we investigated the genetic polymorphisms of the *SPRN* gene in Hanwoo and Holstein cattle. A total of seven polymorphisms, including one novel SNP, were identified in Korean cattle. Notably, genotype, allele and haplotype frequencies were significantly different between Hanwoo and Holstein cattle (Tables 1, 3).

Next, we compared the genotype and allele frequencies of c.199_210delGCCGCGGCGGGG to evaluate the susceptibility of atypical BSE in Korean cattle. Notably, the genotype and allele distributions of c.199_210delGCCGCGGCGGGG in Hanwoo cattle were similar to those of Polish atypical BSE-affected cattle. In addition, the genotype and allele distributions of c.199_210delGCCGCGGCGGGG of Holstein cattle were significantly different from those of Polish atypical BSE-affected cattle (Fig. 2). These results suggest that in based on the genotype and allele distributions of the c.199_210delGCCGCGGCGGGG polymorphism, Hanwoo cattle shows more potential susceptible genetic characteristics in the *SPRN* gene compared to Holstein cattle. However, although the significance of distributional similarity and/or differences of indel polymorphisms between Korean cattle and atypical BSE-affected cattle has been identified in a statistical manner, a low number of BSE-related polymorphisms were observed in atypical BSE-affected cattle. Further confirmation of the association between indel polymorphism and the susceptibility to atypical BSE in a larger population is needed. Furthermore, previous studies have reported indel polymorphism in some healthy animals of *Bos taurus*, *Bos gaurus* (gaur, EU605794.1), and *Bison bison* (American bison, HM179105.1, HM179104.1). It is also possible that the distributional difference in the indel polymorphism could be related to breed origin rather than to the disease. Since the association study has been limited to only Korean commercial breeds and healthy animals, further investigation of several cattle breeds in countries where atypical BSE has been reported in a larger population is needed to clarify the association of the indel polymorphism with susceptibility to atypical BSE in the future.

Next, we estimated the deleterious effects of polymorphisms causing protein sequence changes to the Sho protein using PROVEAN. Notably, the c.110G>C (G37A) and c.199_210delGCCGCGGCGGGG (67_70delAAAG) polymorphisms were predicted as “deleterious”. Using PROVEAN, we found additional genetic factors that can influence the protein structure or function. The *SPRN* gene was expressed dominantly in the brain and overlapped with the expression profile of the *PRNP* gene. In addition, both genes coregulate the gene expression of each other. The potential role of the Sho protein in TSE pathogenesis was demonstrated by the dramatic reduction of the Sho protein in the brain of RML scrapie-infected mice^33^. In addition, the hydrophobic sequence AGAAAGA of the Sho protein has been shown to be crucial for PrP^C^-PrP^Sc^ interaction^34^. In addition, several polymorphisms in the coding region of the *SPRN* gene were significantly associated with the susceptibility of various TSEs. To clarify the effect of a novel candidate gene factor, G37A, in the pathogenesis of TSE, in vivo or in vitro experiments need to be applied to these polymorphisms.

In conclusion, for the first time, we investigated the genotype, allele and haplotype frequencies of bovine *SPRN* polymorphisms in Korean cattle and the significantly different distributions of genotype, allele and haplotype frequencies of bovine *SPRN* polymorphisms between Hanwoo and Holstein cattle. We also found that Hanwoo cattle showed more potential susceptible genetic features of the *SPRN* gene than Holstein cattle using comparisons of genotype and allele distributions of a 12-bp insertion/deletion polymorphism found in atypical BSE-infected Polish cattle. Finally, we estimated the impact of polymorphisms on the Sho protein using an in silico analysis and found a novel candidate SNP, G37A, which can induce structural or functional effects on the Sho protein. To the best of our knowledge, this is the first report of the *SPRN* gene in Korean cattle.

## Methods

### Genomic DNA extraction

Blood samples of 235 Hanwoo and 212 Holstein cattle were obtained in ethylenediaminetetraacetic acid (EDTA) tubes. Genomic DNA was purified from 200 μl peripheral blood using the QIAamp DNA Blood Mini Kit (Qiagen, Valencia California, USA) following the supplier’s instructions.

### Genetic analysis

Polymerase chain reaction (PCR) was performed to amplify the bovine *SPRN* gene with the following gene-specific primers: Bovine *SPRN*-Forward (5′-TGAGATCTCCTTCTCCGTCC-3′) and Bovine *SPRN*-Reverse (5′-GAGGTGTCACAGCTTCAGG-3′). The primers were designed based on the genomic sequence of the bovine *SPRN* gene registered in GenBank (Gene ID: JQ811202.1). The PCR mixture contained 10 μM each primer, 2.5 μl of 10 X *Taq* DNA polymerase reaction buffer containing 25 mM MgCl_2_, 2.5 mM dNTP mixture, 5X Band Doctor™ and 2.5 units of DiaStar™ *EF Taq* DNA polymerase (SolGent, Daejeon, Republic of Korea). The PCR was carried out as follows: predenaturation at 98 °C for 2 min, 33 cycles of denaturation at 98 °C for 20 s, annealing primers at 56 °C for 30 s, extension at 72 °C for 1 min, and final extension at 72 °C for 5 min. The purification of PCR products for sequencing analysis was performed with a QIAquick Gel Extraction Kit (Qiagen, Valencia, California, USA). The PCR products were directly sequenced by an ABI 3730XL sequencer (Applied Biosystems, Foster City, California, USA). The sequencing quality of 628 bp lengths on amplicons of bovine *SPRN* gene was checked by phred quality score using CodonCode Aligner 9.0.1 (CodonCode Corporation, Unitied Kingdom) and genotyping of each nucleotide with Q > 40 was performed using Finch TV software (Geospiza Inc, Seattle, USA). Analyses of LD and haplotype distributions of Hanwoo and Korean Holstein cattle were performed using Haploview 4.2 software. Hardy–Weinberg equilibrium (HWE), genotype, allele and haplotype frequencies were compared by chi-square test (χ^2^) and Fisher’s exact test using SAS 9.4 Software (SAS Institute Inc., Cary, NC, USA).

### Evaluation of the deleterious effect of polymorphisms causing changes in the protein sequence

The PROVEAN program was used to evaluate the biological impact of polymorphisms on the protein function and structure. The PROVEAN scores are computed based on the homologs collected from the NCBI database. The top 30 clusters of closely related sequences from the supporting sequence set were used for evaluations. Score predictions have two types of thresholds; below − 2.5 are considered “deleterious”, and above − 2.5 are considered “neutral”.

### Ethical statement

All experimental procedures were approved by the Jeonbuk National University Institutional Animal Care and Use Committee (IACUC number: CBNU 2018-0079). All experiments using Korean native cattle (Hanwoo) and Korean Holstein cattle were performed in accordance with the Korea Experimental Animal Protection Act.

## Supplementary information

Supplementary Figures.
