# Supplementary material for: Polymorphisms of shadow of prion protein gene (SPRN) in Korean native cattle (Hanwoo) and Holstein cattle
Source: Sci Rep. 2020 Sep 17;10:15272. doi: 10.1038/s41598-020-72225-x (PMC7499179; doi:10.1038/s41598-020-72225-x)
Supplement: Supplementary file 1 — Supplementary Figures. [file 41598_2020_72225_MOESM1_ESM.pdf]

Polymorphisms of shadow of prion protein gene (*SPRN*) in Korean native cattle (Hanwoo) and Holstein cattle

Yong-Chan Kim[1, 2], Seon-Kwan Kim[1, 2], Sae-Young Won[1, 2], Byung-Hoon Jeong[1, 2*]

[1]Korea Zoonosis Research Institute, Jeonbuk National University, Iksan, 570-390, Republic of Korea

[2]Department of Bioactive Material Sciences and Institute for Molecular Biology and Genetics, Jeonbuk National University, Jeonju, 561-756, Republic of Korea

*** Corresponding author:**

Byung-Hoon Jeong, Ph. D,

Korea Zoonosis Research Institute, Jeonbuk National University,

820-120, Hana-ro, Iksan, Jeonbuk 570-390, Republic of Korea.

TEL: 82-63-900-4040, FAX: 82-63-900-4012, E-mail: bhjeong@jbnu.ac.kr

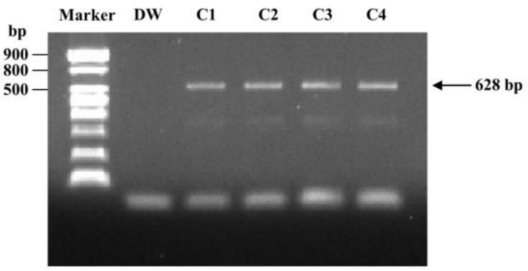
Marker
DW
C1
C2
C3
C4
bp
900
800
500
628 bp

Supplementary Figure 1 Amplification of the shadow of prion protein gene (*SPRN*) in cattle. Amplicons of the bovine *SPRN* gene using gene-specific primers were detected at 628 bp from 4 different individuals of cattle. Marker: 100bp DNA marker; DW: negative control for polymerase chain reaction (PCR). The PCR reactions for negative control were performed with PCR-grade distilled water as a template. Except for template, the composition of PCR reagents for negative control was equal to that of other lanes; lane 3-6: amplicons of bovine *SPRN* gene from each individual. Full-length gels are presented in Supplementary Figure 2.

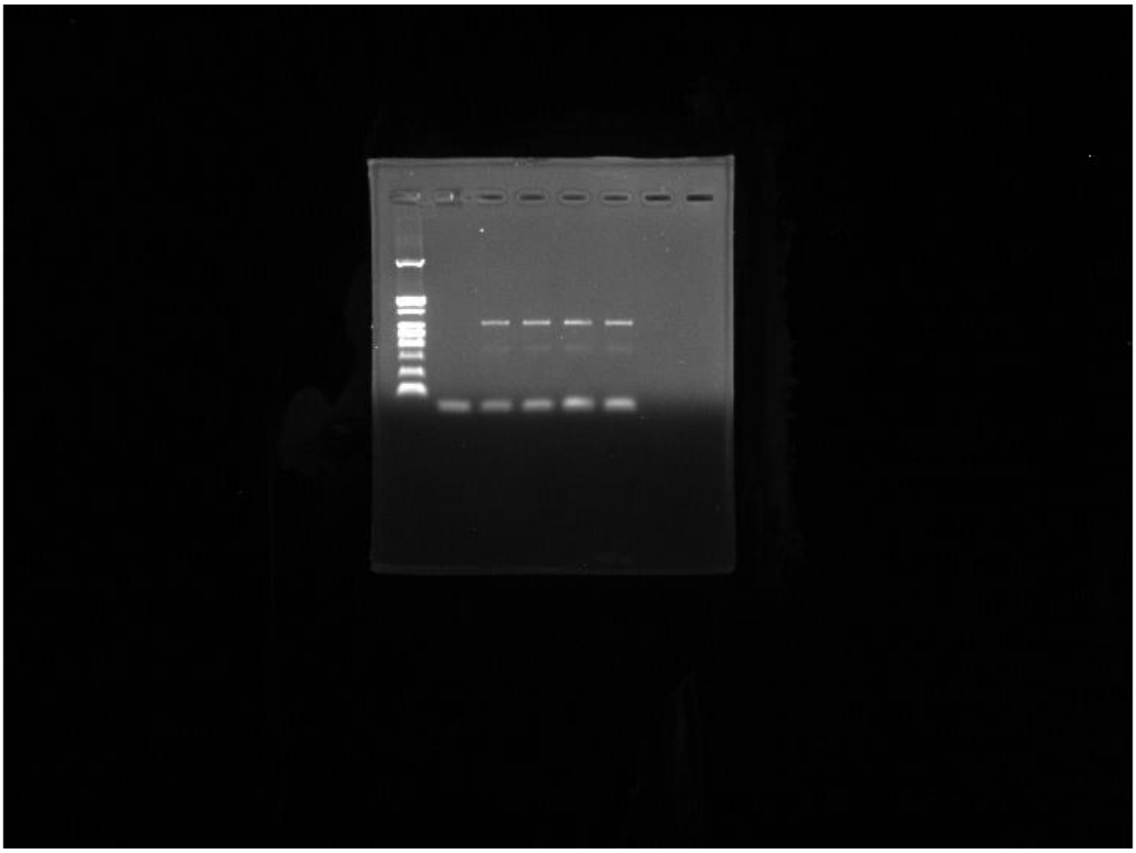

Supplementary Figure 2 Full-length gels image of Supplementary Figure 1.

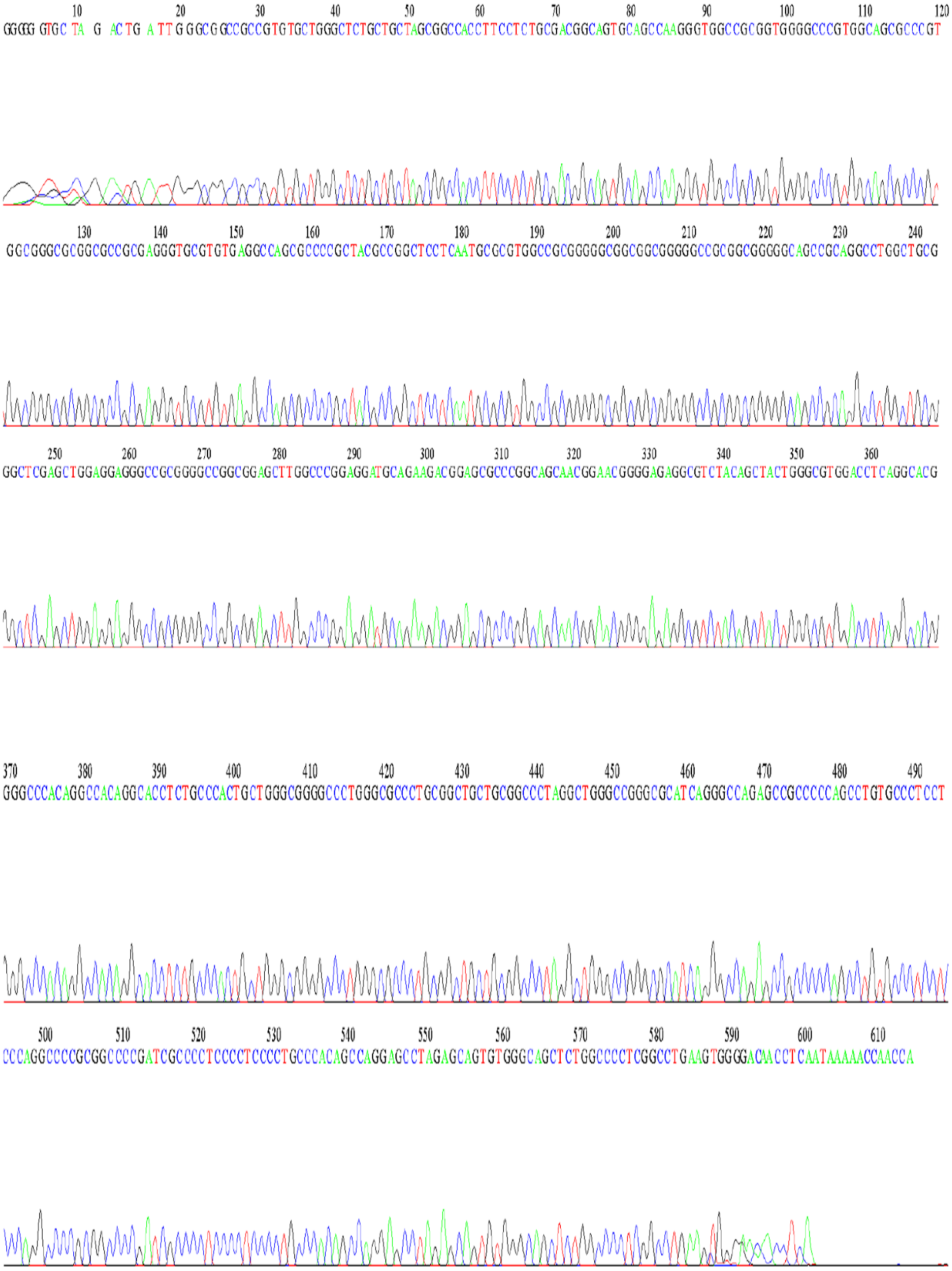

Supplementary Figure 3 Representative image of sequencing results on amplicon of bovine *SPRN* gene.

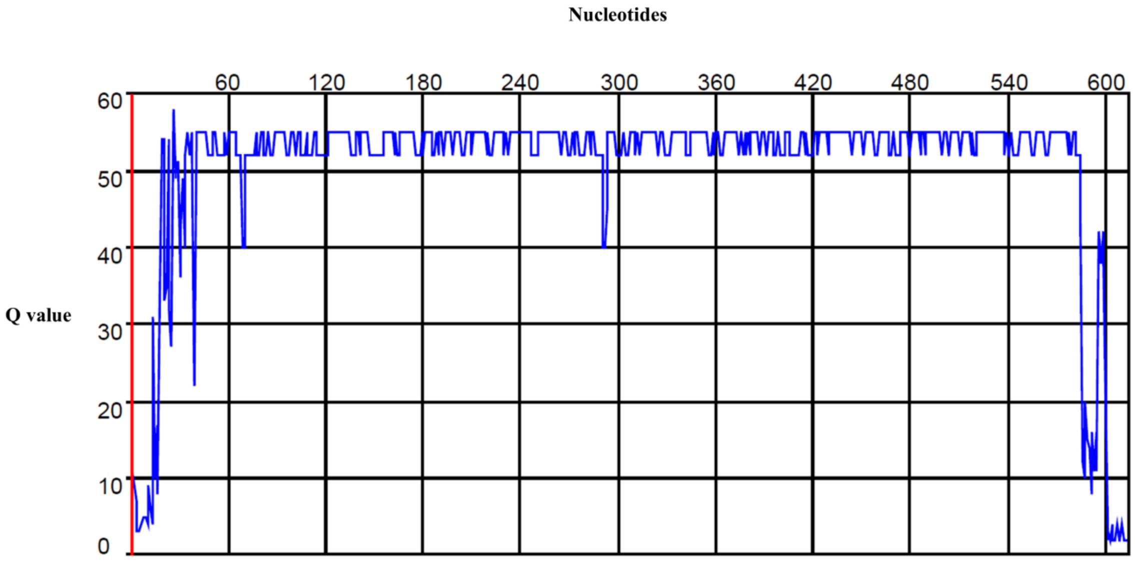

Nucleotides
60
120
180
240
300
360
420
480
540
600
Q value
60
50
40
30
20
10
0

Supplementary Figure 4 Representative results on quality check of sequencing results performed with amplicons of bovine *SPRN* gene. X axis: Q value; Y axis: nucleotides.
